# Shared Medical Appointments May Be Effective for Improving Clinical and Behavioral Outcomes in Type 2 Diabetes: A Narrative Review

**DOI:** 10.3389/fendo.2017.00263

**Published:** 2017-10-04

**Authors:** Kirthi Menon, Aya Mousa, Maximilian PJ de Courten, Georgia Soldatos, Garry Egger, Barbora de Courten

**Affiliations:** ^1^Monash Centre for Health Research and Implementation, School of Public Health and Preventive Medicine, Monash University, Melbourne, VIC, Australia; ^2^Centre for Chronic Disease, Victoria University, Australia, Melbourne, VIC, Australia; ^3^Centre for Health Promotion and Research, Health and Human Sciences Department, Southern Cross University, Lismore, NSW, Australia

**Keywords:** shared medical appointments, group visits, type 2 diabetes, clinical care, model of care

## Abstract

Type 2 diabetes mellitus (T2DM) is a complex chronic disease affecting over 400 million people worldwide. Managing T2DM and its associated complications in individual patient consultations poses substantial challenges to physicians due to limited time and resources and lack of access to multidisciplinary teams. Shared medical appointments (SMAs) are consecutive medical consultations provided by a physician in a group setting, where integrated medical care and patient education are delivered in a single session. SMAs allow physicians to deliver the same level of care to multiple patients at the same time, thereby maximizing available resources. However, the effectiveness and practicality of SMAs in the management of T2DM remains unknown. This narrative review summarizes current and emerging evidence regarding the effectiveness of SMAs in improving clinical outcomes in patients with T2DM, as well as whether SMAs are associated with reduced costs and improved diabetes-related behavioral and lifestyle changes. An extensive literature search was conducted on major electronic databases including PubMed and Google Scholar using keywords, including SMAs, group visits, and T2DM to identify all studies of SMAs in patients with T2DM. Studies in type 1 diabetes or mixed or unspecified populations were excluded, as well as studies where SMAs did not involve a physician since these do not meet the classical definition of a SMA. Nineteen studies were identified and are included in this review. Overall, current evidence suggests that SMAs delivered regularly over time may be effective in improving glycemic outcomes, diabetes knowledge, and some diabetes-related behaviors. However, the main limitation of existing studies was the paucity of comparisons with standard care which limits the ability to draw conclusions regarding whether SMAs are superior to standard care in T2DM management. Moreover, the small number of studies and substantial heterogeneity in study designs, populations, and interventions creates difficulties in establishing the practicality and efficiency of SMAs in the clinical care setting. We conclude that there remains a need for larger studies to identify populations who may or may not benefit from the SMA model of care and to clarify the potential benefits and barriers to implementing SMAs into routine diabetes care.

## Introduction

Type 2 diabetes mellitus (T2DM) is a leading cause of morbidity and mortality, affecting 422 million adults globally (1). The prevalence of T2DM is rising in line with increased obesity (2), which today affects >1.9 billion adults worldwide (3). T2DM and its microvascular (retinopathy, nephropathy, neuropathy) and macrovascular (cardiovascular, cerebrovascular, peripheral vascular) complications pose a substantial health and cost burden, accounting for >2 million deaths each year (4) and costing >$827 billion in direct costs annually (5). In view of the current T2DM burden, it is imperative to identify innovative and integrated models of care in order to improve disease management in clinical settings.

Currently, complex chronic diseases such as T2DM are managed through individual patient–doctor consultations (6). However, delivering high-quality evidence-based care in a one-on-one setting is challenging due to the increasing number of patients with complex diseases, limited time for consultations, limited resources, and lack of access to multidisciplinary teams or integrated models of care (6). There is also no convincing evidence of the comparative effectiveness or otherwise of the one-on-one approach over any other. One approach which has been proposed to address these challenges is shared medical appointments (SMAs).

Originally developed by Caballero (6), SMAs are defined as: “…*consecutive individual medical visits carried out in a supportive group setting of similar patients where all can listen, interact, and learn*.” SMAs are group or cluster medical consultations (7), carried out sequentially, where approximately 8–15 patients with the same chronic condition are seen by a physician and an interdisciplinary team of health-care professionals over a 60- to 120-min time period (8). This allows physicians to deliver the same level of care to multiple patients at the same time, thereby maximizing available resources (6). SMAs must involve a general practitioner or physician (GP) and are typically facilitated by a nurse practitioner or other allied health professional (9). SMAs, therefore, differ from disease-specific support groups or group education sessions in that provision of medical care by a qualified physician (including diagnostic and laboratory assessments, prescriptions, and medical reviews, treatment, and management) is provided within the SMA care paradigm (10). Following the physician consultation, patients may engage with nurses or allied health professionals to discuss various topics related to their chronic disease, and an educational component is often held as a talk, discussion, or audio-visual presentation, generally without the doctor present (6). The concept of SMAs is based on providing integrated medical care and patient education, making it an ideal format for managing complex diseases in a primary care setting, with some SMAs also being conducted in hospitals (10, 11).

As with any new model of care, introducing SMAs into routine clinical care may encounter challenges and may have unanticipated negative effects. For instance, SMAs may introduce concerns around patient confidentiality or preference to visit their regular physician, as well as issues around the logistics of setting up SMAs, including availability of suitable spaces and materials and availability of trained staff with specific item number reimbursements (9, 12). It is also unknown whether SMAs may be more costly to the health-care system or whether they could have adverse effects on clinical outcomes or patient and/or physician satisfaction. Most importantly, the potential value of SMAs over any other form of clinical interaction in patients with T2DM has not been established and is a key first step toward their successful integration into routine diabetes care.

This review synthesizes current and emerging evidence on the effectiveness of SMAs in the management of T2DM and identifies relevant knowledge gaps. Specifically, we aimed to establish whether SMAs improved clinical outcomes compared to usual care, their acceptability among patients and providers, and whether they were associated with reduced costs or improved diabetes-related behavioral and lifestyle changes.

## Materials and Methods

Major electronic databases including PubMed and Google Scholar were searched to identify studies of SMAs in patients with T2DM. The following keywords and phrases were used in various combinations: SMA, group medical appointments, group care, group visits, group-based education, T2DM. All publications from inception up to the when the search was conducted (February 2017) were considered. All articles identified within the initial search were screened for relevance and content by two independent reviewers (Kirthi Menon and Aya Mousa), and their bibliographies were searched for any additional relevant articles.

Criteria for inclusion were articles reporting observational studies or randomized or quasi-randomized trials where SMAs were delivered in any frequency and for any duration to patients with T2DM. Studies of SMAs in patients with type 1 diabetes or mixed or unspecified populations were excluded, as well as studies where SMAs did not involve a physician since these do not meet the classical definition of a SMA. The review covers retrospective, prospective, and randomized controlled studies in a narrative review format, and is not intended as a systematic review.

## Retrospective Studies

### Study and Sample Characteristics

Three retrospective studies (10, 13, 14) were identified examining the effectiveness of SMAs on T2DM-related outcomes. Details of these studies are outlined in Table 1 (A). All three studies were conducted in the US and utilized medical records to compare patients who attended SMAs to a control group of patients who received usual care. One study by Culhane-Pera et al. (14) included three groups: an SMA group (*n* = 39), a control group derived from medical records (*n* = 216), and an additional comparison group of patients who declined participation in the SMAs (*n* = 22). There were no differences in demographic or clinical characteristics between SMA participants and those who declined participation in this study. Total sample sizes ranged from 160 (13) to 988 (10) participants, and total numbers of participants attending SMAs over the study duration ranged from 39 (14) to 371 (10) participants. Medical record data were extracted for periods of 12 (13) and 13 months (14) and 3 years (10). However, the 3-year study by Harris et al. (10) did not report the numbers, frequencies, or duration of SMAs attended, specifying only that patients were included if they attended at least one SMA annually for two consecutive years. In the two remaining studies, patients attended four 120-min SMA sessions over 6 months (13) or seven 210-min SMAs over 13 months (14). The number of participants was 3–12 (13) and 10–16 (14) per SMA session (Table 1, A). Interventions delivered as part of the SMAs included a group education component in two studies (13, 14), while the third did not specify (10). All three studies were targeted at specific population groups which included: an older Veteran population (10); a rural minority group of primarily African-Americans (13); and a group of refugees from the Vietnam War known as Hmong people (originating from Southern China) (14). The study by Bray et al. (13) specifically recruited high-risk patients with HbA1c >7%, blood pressure >135/85 mmHg, or physical or laboratory evidence of end-organ complications, including retinopathy, neuropathy, or nephropathy.

**Table 1:** Characteristics of studies in a review of physician-based SMAs in patients with type 2 diabetes.

Reference, country	Study design and setting	Study duration (months); SMAs attended (*n*); frequency (*n*); duration (min); patients per SMA session (*n*)	Comparator	*n*	Mean age (years); sex, %; ethnicity (%)	Mean BMI (kg/m^2^); T2DM duration (years); Smokers (*n*)	Clinical outcomes measured	Main findings for effect of SMAs
**(A) Retrospective studies**								
Harris et al., USA (10)	Retrospective (abstracted medical records); large midwestern veterans administration hospital	36 months (3 years); NR (minimum 2 years of attending SMAs); Duration NR; *n* per SMA NR	Usual care (medical records)	*n* = 988 *I*: 371 *C*: 617	Age: 70.1 Male: 98% Ethnicity: White (72); Black (20)	BMI: NR (53.9% obese) Duration: NR Current smoker: 413	HbA1c, SBP, LDL	NS

Culhane-Pera et al., USA (14)	Retrospective (diabetes registry medical records) with controls; federally qualified community health center	13 months; 7 SMAs (monthly for 3 months then quarterly); 210 min; *n* = 10–16; median 7 per SMA	C_1_ = usual care; C_2_ = refused to participate (both medical records)	*n* = 277 *I*: 39 *C*_1_: 238 *C*_2_: 22	Age: 56.8 Male: 36% Ethnicity: Hmong (originating in Southern China)	BMI: 28.5 Duration: 6.6 Smoker: NR	BMI, HbA1c, SBP, DBP, LDL, HDL, TC, TRIG, microalbumin/creatinine ratio	HbA1c: NS; Other outcomes only assessed for within group difference: NS

Bray et al., USA (13)	Retrospective (database medical records) with controls; Rural fee-for-service primary care practices	12 months; 4 SMAs over 6 months; 120 min; *n* = 3–12 per SMA	Usual care (medical records)	*n* = 160 *I*: 112 *C*: 48	Age: 59.4 Male: 44.4% Ethnicity: >90% Black	BMI: NR Duration: NR Smoker: NR	Weight, HbA1c, BP	↓HbA1c

**(B) Prospective studies**								
Boegner et al., France (15)	Prospective pre-test/post-test design; multidisciplinary private practice settings (GPs, specialists, etc.)	6 months; 2 SMAs (frequency NR); Half-day; Mean *n* = 4 per SMA	No control group/comparator	n = 427 I: 322 C: N/A	Age: 64.6 ± 10.0 Male: 55.3% Ethnicity: NR	BMI: 28.9 Duration: 11.5 ± 12.2 Current smoker: 26	Weight; HbA1c; FBG; SBP; DBP	↓HbA1c; ↓FBG

Dickman et al., USA (16)	Prospective pre-test/post-test design; Free primary care clinic	4 months; 4 SMAs (monthly)—80% of patients attended 4 SMAs; 90 min; *n* = 8–12 per SMA	No control group/comparator	*n* = 37 *I*: 30 *C*: N/A	Age: 57.0 ± 10.2 Male: 34% Ethnicity: Hispanic (61); Asian (11); Middle Eastern (11); Black (8.5); White (8.5)	BMI: NR Duration: NR Smoker: NR	Weight, HbA1c, SBP, LDL	SBP: NS Remaining outcomes: NR

Guthrie and Bogue, USA (17)	Prospective pre-test/post-test design; Community family medicine residency practice	2 months; 16 SMAs offered (NR attended—patients advised to attend as many as possible); 60–90 min *n* per SMA NR	No control group/comparator	*n* = 48 *I*: 46 *C*: N/A	Age: 64.8; Male: 39%; Ethnicity: White (67); Black (24); Hispanic (4); Asian/Pacific Islander (4);	BMI: 34.5 Duration: NR Smoker: NR	Weight, TC, LDL, HDL, TRIG, HDL/TC ratio	↓Weight

Palaniappan et al. USA (11)	Prospective with a created (matched) control group; Primary care clinic at research institute-affiliated hospital	24 months; Mean of 3 SMAs attended over 4–5 months (offered biweekly); median of 6 weeks between visits; 90 min; *n* = 6–12 per SMA	Matched controls receiving usual office visit with same physician	*n* = 430 *I*: 74 *C*: 356	Age: 49.7; Male: 34%; Ethnicity: NR	BMI: 32.4; Duration: NR; Smoker: NR	Weight, BMI	↓Weight; ↓BMI between groups and associated with total SMAs attended

Pieber et al., Austria (18)	Prospective with concurrent non-randomized controls; Rural primary care clinics	6 months; 4 SMAs (weekly); 90–120 min; *n* = 4–8 per SMA	Usual care (medical records from other GPs prior to initiating SMAs)	*n* = 94 *I*: 45 *C*: 49	Age: 64.7 Male: 45% Ethnicity: NR	BMI: 30.2; Duration: 7.3; Smoker: NR	Weight, BMI, HbA1c; SBP, DBP, TC, TRIG	↓Weight; ↓BMI; ↓HbA1c; ↓DBP; ↓TRIG;TC and SBP: NS

Kirsh et al., USA (8)	Prospective with concurrent non-randomized controls; Primary care clinic tertiary academic medical center—Veteran Affairs health system	4 months; Drop-ins: 1–7 SMAS over 4 months (39% attended 1 SMA; 77% attended 1–3 SMAs); 60–120 min; *n* = up to 8 per SMA	Usual care (medical records from other GPs prior to initiating SMAs)	*n* = 79 *I*: 44 *C*: 35	Age: 60.6 Male: 97.7% Ethnicity: NR	BMI: NR Duration: NR Smoker: NR	HbA1c, LDL, SBP	↓HbA1c; ↓SBP

**(C) RCTs**								
Clancy et al., USA (22)	RCT; primary care university affiliated clinic	6 months; 6 SMAs (monthly); 120 min; *n* = 19–20 per SMA	Usual care (details NR)	*n* = 120 *I*: 59 *C*: 61	Age: 54; Male: 22%; Ethnicity: Black (77.5)	BMI: NR; Duration: NR; Smoker: NR	HbA1c, TC, HDL, LDL	NS

Clancy et al., USA (27, 28)	RCT; Primary care university affiliated clinic	12 months; 12 SMAs (monthly) 120 min; *n* = 14–17 per SMA	Usual care (details NR)	*n* = 186 *I*: 96 *C*: 90	Age: 56; Male: 28%; Ethnicity: Black (83.3)	BMI: NR; Duration: NR; Current Smoker: *I*: 20; *C*: 19	HbA1c, TC, LDL, HDL, TRIG	NS

Gutierrez et al., USA (23)	RCT; Primary care university affiliated clinic	17 months; 36 SMAs offered (biweekly), actual SMAs attended NR; Duration NR; Mean *n* = 9 per SMA	Usual care (details NR)	*n* = 103 *I*: 50 *C*: 53	Age: NR; Male: NR; Ethnicity: Hispanic (100)	BMI: NR; Duration: NR; Smoker: NR	HbA1c	↓HbA1c

Trento et al., Italy (2- year follow up) (25)	RCT; Diabetes university affiliated clinic	24 months; 7–8 offered (quarterly); 120 min; *n* = 9–10 per SMA	Usual care (7–8 attended quarterly with individual education sessions)	*n* = 112 *I*: 56 *C*: 56	Age: 61.5; Male: 54%; Ethnicity: NR	BMI: *I*: 29.7 ± 4.5; *C*: 27.8 ± 4.1; Median (range) duration: *I*: 9.4 (1–23); *C*: 9.8 (1–39); Current Smoker: *I*: 10; *C*: 15	Weight, BMI, HbA1c, FBG, TC, HDL, TRIG, creatinine, albuminuria	↓HbA1c, ↑HDL, Trend for ↓BMI and ↓TRIG in SMA group.
Trento et al., 2004, Italy (5-year follow up) (20)		60 months (5 years); 19 SMAs offered (quarterly); 120 min; *n* = 9–10 per SMA		*n* = 84 *I*: 42 *C*: 42				↓Weight; ↓HbA1c Trend for ↓BMI in SMA group.

Trento et al., Italy (21)	RCT; diabetes university affiliated clinic	48 months (4 years) 14 SMAs (quarterly); 60 min; *n* = 9–10 per SMA	Usual care (14 attended quarterly)	*n* = 581 *I*: 315 *C*: 266	Age: 69.3 ± 8.4; Male: 51%; Ethnicity: NR	BMI: *I*: 30.6 ± 4.9; *C*: 29.3 ± 5.1; Median duration: *I*: 15.7 ± 6.9; *C*: 16.6 ± 7.2; Current Smoker: *I*: 79; *C*: 67	Weight, BMI, HbA1c, FBG, TC, HDL, LDL, TRIG, SBP, DBP, creatinine	↓Weight, ↓BMI, ↓FBG, ↓HbA1c, ↓TC, ↓LDL, ↓TRIG, ↓SBP, ↓DBP, and ↑HDL

Rygg et al., Norway (24)	Open pragmatic RCT	12 months; 3 SMAs (biweekly for 6 weeks or triweekly for 9 weeks) 300 min; median *n* = 4	Usual care (details NR)	*n* = 146 *I*: 73 *C*: 73	Age: 66; Male: 53%; Ethnicity: Norwegian-White (100)	BMI: *I*: 30.0 ± 4.3; *C*: 30.7 ± 5.5; Median (IQR) duration: All: 5 (2.5–10); Smoker: NR	Weight, BMI, HbA1c, TC, HDL, TRIG, SBP, DBP, Creatinine	NS

Naik et al., USA (26)	RCT; primary care clinic—Veteran Affairs health system	12 months; 4 SMAs (triweekly over 3 months) 60 min; *n* = 5–7 per SMA	Enhanced usual care (x2 attended with 120 min diabetes education session preceding each usual care visit)	*n* = 85 *I*: 44 *C*: 41	Age: 63.6 ± 7.9; Male: NR; Ethnicity: Black (31)	BMI: *I*: 33.4 ± 6.5; *C*: 34.2 ± 6.7; Duration: *I*: 4.98 ± 3.1; *C*: 5.04 ± 3.0; Smoker: NR	HbA1c	↓HbA1c at 3 months; trend toward ↓HbA1c at 1 year

Schillinger et al., USA (19)	3-arm RCT; Country-run university- affiliated primary care clinics	9 months; 9 SMAs (monthly)—mean of 4.8 SMAs attended 90 min; *n* = 6–10 per SMA	*C*_1_ = usual care (details NR); *C*_2_ = weekly interactive automated telephone support + nurse follow up	*n* = 339 *I*: 113 *C*_1_: 114 *C*_2_: 112	Age: 56.1 ± 12.0; Male: 41%; Ethnicity: Asian (23.3); Black (20.6); White/Latino (46.9); White/non-Latino (7.7); other (1.5)	BMI: 31.5 ± 10.0; *I*: 31.9 ± 8.2; *C*_1_: 32.3 ± 13.5; *C*_2_: 30.3 ± 6.7; Duration: 9.5 ± 7.4; I: 9.2 ± 6.8; *C*_1_: 10.4 ± 8.1; *C*_2_: 9.1 ± 7.3; Smoker: NR	BMI; HbA1c; SBP; DBP	NS

*Data are reported as mean ± SD, %, or n, unless otherwise specified*.

*SMA, shared medical appointments; RCT, randomized controlled trial; T2DM, type 2 diabetes mellitus; I, intervention; C, control; NR, not reported; N/A, not applicable; NS, not significant; BMI, body mass index; HbA1c, glycated hemoglobin; TC, total cholesterol; HDL/LDL, high-density/low-density lipoprotein cholesterol; TRIG, triglycerides; FBG, fasting blood glucose; SBP/DBP, systolic/diastolic blood pressure; CVD, cardiovascular disease*.

### Clinical Outcomes

Clinical outcomes reported included anthropometry (weight and/or BMI; *n* = 2), glycated hemoglobin (HbA1c; *n* = 3), blood pressure (*n* = 3), and serum lipids (*n* = 2), including total cholesterol, high- and low-density lipoprotein cholesterol (HDL; LDL), and triglycerides. Culhane-Pera et al. (14) also measured microalbumin/creatinine ratio. In the two studies reporting anthropometric outcomes (13, 14), weight and BMI did not differ significantly between patients who attended four to seven SMAs over 6–13 months and those who received usual care. Similarly, no differences were observed in blood pressure, LDL, or microalbumin/creatinine ratio between SMA and usual care patients in any of the studies (10, 13, 14). Only the study in African-Americans (13) reported improvements in HbA1c, from 8.2 to 7.1% in those who attended four SMAs over 6 months versus usual care (8.3–8.6%). Note that this was the only study which specifically recruited high-risk patients with HbA1c > 7%, and the SMA was comparatively intensive with 2-h SMAs attended four times over 6 months (13). Thus, it is possible that SMAs may be more effective when delivered frequently to patients with poor glycemic control. The lack of differences reported between SMA and control groups for most clinical outcomes may be due to the retrospective nature of these studies, since they were not designed specifically for these outcomes. Alternatively, it is possible that SMAs were not superior to standard care in improving clinical outcomes in these studies, or that they may only be effective in certain subgroups of patients; however, drawing conclusions regarding specific subgroups is limited by the diverse demographic, socioeconomic, and cultural characteristics of the study populations.

### Other Outcomes

Harris et al. (10) examined health-care utilization and found no differences in emergency department (ED) visits; however, the mean total health-care visits at study-end was 28 in SMA participants compared to 19 in patients receiving usual care (10). This was likely because this study had a high-risk SMA cohort who had a higher proportion of smoking, alcoholism, substance abuse, depression, etc., as well as higher ED and total health-care visits at baseline, compared to the usual care group. None of these baseline differences were adjusted for in the analyses, thus results should be interpreted with caution.

Culhane-Pera et al. (14) investigated changes in behavioral outcomes (24-h diet and exercise recall), and mental health outcomes (depression and anxiety scores on Hmong Likert scale survey) pre- and post-SMAs. These were not compared between groups due to unavailable control group data. After seven SMAs over 13 months, total anxiety-depression scores as well as anxiety-subscale scores were improved, with no change in depression-subscale scores (14). There were no changes observed in dietary or exercise habits in SMA participants. These analyses involved only 39 low-income, elderly Hmong refugees, hence interpretation of these findings is limited. The lack of changes observed in diet and exercise habits in this study may also be due to difficulties associated with lifestyle changes, which often require regular contact and monitoring, and thus may depend more on the frequency of contact rather than on the model of care delivered. Overall, conclusions on SMA effectiveness are difficult to draw from this group of studies.

## Prospective Studies

### Study and Sample Characteristics

Six prospective studies (8, 11, 15–18) of SMAs in T2DM were identified and are detailed in Table 1 (B). All studies were conducted in the US (*n* = 4) or Europe (France, *n* = 1; Austria, *n* = 1) (Table 1, B). Three of the six studies were of a pretest/posttest design while the remaining three compared SMAs with a control group created by using data from medical records of patients attending usual care. Sample sizes varied considerably with total numbers of participants ranging from 37 (*n* = 30 analyzed) (16) to 430 participants (11). However, in studies which had both an SMA group and a control group (*n* = 3) (8, 11, 18), the largest number of participants in the SMA group was 74 (11). Study durations and numbers of SMAs attended were limited, with most studies having between two and four SMAs over 4–6 months. One study by Guthrie and Bogue (17) offered 16 SMAs over 2 months but did not report the number of SMAs attended, while Kirsh et al. offered drop-ins (8) and reported that 77% of participants attended one to three SMA sessions over 4 months. All studies reported SMA sessions of between 60 and 120 min, with the exception of one study (15) which conducted half-day long SMAs. The number of participants in each SMA ranged between 4 (15) and 8–12 (16) per session, and one study (17) did not report the number of participants per session.

The types of interventions delivered within the SMAs varied across studies; however, all included an educational component (15–18) or discussion of relevant health-related topics and concerns (8, 11), in addition to the individual patient or medication reviews (8, 16, 18). In the three studies which included a control group, two formed the control group using medical records of patients attending GPs who had not yet commenced SMA sessions within their practices (8, 18). The third study used medical records of patients attending individual visits with the same physician who performed the SMAs, where patients were included as controls if they had a similar age, sex, BMI, and weight as patients in the SMA group (11). Some studies targeted specific population groups including Veterans at high risk of cardiovascular disease (CVD) (HbA1c > 9%; systolic blood pressure (SBP) >160 mmHg; and LDL >130 mg/dL) (8) or low-income, uninsured, and/or unemployed groups (16, 18) (Table 1, B). Participant HbA1c level was as an inclusion criterion in the study of Veterans at high-risk of CVD (8) as noted above (HbA1c > 9%), while another study (16) specified a HbA1c < 9% in their inclusion criteria.

### Clinical Outcomes

Clinical outcomes included weight and/or BMI, fasting blood glucose (FBG), HbA1c, blood pressure, and serum lipids (Table 1, B). Five studies (11, 15–18) measured weight and/or BMI as an outcome, three of which reported significant findings. Palaniappan et al. (11) and Pieber et al. (18) reported reduced weight (−0.5 and −2.7 kg, respectively) and BMI (−0.3 and −1.1 kg/m^2^, respectively) in those who attended three to four SMAs over 4–6 months, compared to no change in controls. The third study by Guthrie and Bogue (17) was a pretest posttest design which reported a weight loss of −4.1 kg within the SMA group, who were offered up to 16 SMAs in 2 months. The studies which found no difference in weight or BMI were in non-obese participants (15), had a small sample size (n = 30) (16), or had infrequent SMAs with a limited duration to observe beneficial effects on weight (two SMAs in 6 months) (15). Because weight-loss strategies often require close supervision and monitoring, these findings suggest that longer durations and more frequent SMAs could be beneficial in promoting weight loss in these patients. This is supported by one study (11) which reported that weight loss and BMI reduction were directly correlated with the total number of SMAs attended. Importantly, the studies by Palaniappan et al. (11) and Pieber et al. (18), both of which reported improved weight loss in the SMA group versus controls, did not specify the number of visits attended by the control groups. Thus, it is possible that SMA attenders were compared to both attenders and non-attenders in usual care, making it difficult to ascertain whether the beneficial effects on weight loss are attributed to the SMA model of care or simply to the increased attendance and monitoring of patients in the SMA groups. Larger studies are needed to establish the effectiveness of SMAs in improving anthropometric outcomes, with sufficient detail of the number and nature of usual care visits attended by the control groups.

Regarding glycemic outcomes, one study measured both FBG and HbA1c and four measured HbA1c only. In a pre-test post-test study by Boegner et al. (15), FBG reduced from 8.1 to 7.9 mmol/L and HbA1c reduced from 7.6 to 7.4% after 6 months in patients who received two half-day SMA sessions. However, there was no control group in this study and although improvements in glycemic control were small, these may have resulted from intensified treatment since many of the SMA patients switched from oral agents to insulin during the study period. In the remaining three studies which examined HbA1c in Veterans at high risk of CVD or in low-income uninsured groups (8, 16, 18), two reported significantly lower HbA1c (−1.4 and −0.5%) in those who attended one to seven SMAs over 4–6 months compared with controls (−0.3 and +0.3%, respectively) (8, 18). The third study in a low-income cohort (16) found no difference in HbA1c pre- and post- SMA in patients attending monthly SMAs for 4 months. It is possible that SMAs were not beneficial for improving HbA1c in this cohort; however this study had no control group and included only 30 participants, hence the null findings should be interpreted in light of the limitations of this study and the potential lack of statistical power.

Blood pressure was measured in four studies with inconsistent results. Kirsh et al. (8) reported lower SBP (−14.8 mmHg) in their high-risk Veteran cohort after receiving one to seven SMAs over 4 months compared to controls (−2.5 mmHg), while Pieber et al. (18) reported that in their low-income cohort, attending four SMAs over 6 months reduced diastolic blood pressure (DBP) by −11.1 mmHg compared to −5.4 mmHg in controls, with no change in SBP. The remaining two studies (15, 16) found no difference between pre- and post- SBP or DBP in patients receiving two to four SMAs over 4–6 months. However, the latter two studies had no control group, and one had a small sample size (16), while the other held only two SMA sessions over 6 months (15), which may explain their lack of findings.

Four studies (8, 16–18) measured serum lipids including total cholesterol (*n* = 2), HDL (*n* = 1), LDL (*n* = 3), and triglycerides (*n* = 2). Only the study by Pieber et al. (18) in a low-income cohort reported a significant decrease in triglycerides (−0.6 mmol/L) in patients who attended four SMAs over 6 months, compared to increased triglycerides in those receiving usual care (+0.2 mmol/L). This was the largest (n = 94 compared to n < 74) and longest (6 months compared to ≤4 months) study measuring lipids, suggesting that studies with larger sample sizes and sufficient follow up durations are needed to observe beneficial effects of SMAs on serum lipids.

### Other Outcomes

Most studies reported several other outcomes which included patient satisfaction, cost-effectiveness of SMAs, and whether SMAs improved adherence to treatment guidelines, diabetes-related knowledge and self-management, and lifestyle habits such as diet and exercise. Patient satisfaction was reported in one study (16) which found that 95% of patients rated SMAs as excellent or very good and stated that they would participate in SMAs again. Only the study by Pieber et al. (18) assessed the potential cost-savings associated with SMAs. Here, the number of patients who switched from oral hypoglycemic (OHG) medications to diet and exercise doubled from 16% to 29% in the SMA group, with no significant change in controls (18–14%). Moreover, daily OHG use in the SMA group reduced from 2.2 to 1.6 tablets per patient per day, compared to an increase in controls from 1.9 to 2.3 tablets (18). The reduction in OHG use was likely due to the significant weight loss and improved glycemic control experienced in the SMA group in this study (mean weight loss of −2.7 kg and change in HbA1c of −0.5% as reported earlier) (18). Nevertheless, as a result of reduced OHG prescriptions, it was estimated that SMAs reduced routine health care costs by UK £33 per patient per year compared to a UK£30 increased cost in usual care patients (18).

Adherence to specific performance targets (HbA1c <9%; SBP <160 mmHg; and LDL <130 mg/dL) was assessed in the study by Kirsh et al. (8) of Veterans at high-risk of CVD. The proportion of patients meeting targets in the SMA group rose by 36, 25, and 14% for HbA1c, SBP, and LDL, respectively, compared to no change for HbA1c and LDL and a decreased adherence to SBP targets (from 27 to 18%) in the control group (8). This may be because SMAs promote a team approach and engage patients in their own self-management, which may improve adherence to targets, particularly in high-risk groups (8).

Diabetes-related knowledge and self-management were assessed in two studies (15, 18). Using a validated 21-item questionnaire, Pieber et al. (18) found that total knowledge score for the SMA cohort increased by +25 points compared to +1 in controls, and 92% of SMA patients reported self-monitoring compared to 82% of controls. Similarly, Boegner et al. (15) reported that diabetes knowledge and self-management improved from pre-SMA scores of 56 and 77% to 72 and 82%, respectively, after 6 months of attending SMAs.

Lifestyle and behavioral outcomes were assessed in two studies (16, 17), both of which had no control group. After 2–4 months of SMAs, self-reported exercise per week increased in both studies (16, 17), with Dickman et al. (16) reporting that exercise increased in conjunction with increased attendance to SMAs. Self-reported changes in diet such as reducing red meat consumption and increasing intake of plant-based foods were also improved after attending SMAs for 2 months (17). Similarly, Dickman et al. (16) found that 97% of patients attending SMAs for 4 months achieved or almost achieved their self-prescribed goals, which included reducing stress and unhealthy food consumption, increasing exercise, and taking prescribed medications.

Hence, while it is difficult to draw conclusions from these studies based on clinical outcomes, there do appear to be significant advantages in SMAs in patient satisfaction, adherence to targets, knowledge, and lifestyle and behavioral outcomes. These need to be examined in more depth in future evaluative studies.

## Randomized Controlled Trials

### Study and Sample Characteristics

Ten articles were identified reporting eight randomized controlled trials (RCTs) of SMAs held exclusively in T2DM patients. Details of these RCTs are outlined in Table 1 (C). All studies were conducted in the US and Europe, and with the exception of one three-arm trial (19), all studies were parallel-design RCTs. Sample sizes varied substantially, ranging from 84 (20) to 581 (21) participants in total, with the largest intervention group comprising a total of 315 participants who attended SMAs over four years (21). Study durations ranged from 6 months (22) to 5 years (20), with three of the eight RCTs being of >12 months duration. However, the numbers of SMAs attended in most studies was ≤8 SMAs over 6 months to 2 years, and one study by Gutierrez et al. (23) offered 36 SMAs over 17 months, but did not report the number of SMAs actually attended. The majority of RCTs conducted SMAs monthly with the most frequent being biweekly (23, 24), while the studies by Trento et al. (20, 21, 25) conducted SMAs every 3 months (quarterly) and compared these with quarterly usual care visits. Most SMA durations ranged from 60 (21, 26) to 120 min (20, 22, 25, 27), with the longest single SMA session lasting 5 h (24). In six of the eight RCTs, SMA sessions were relatively small, involving 4–10 patients, while the two studies by Clancy et al. (22, 27) had 14–17 and 19–20 patients per SMA (Table 1, C).

Regarding the interventions provided within the SMAs, all studies involved group education sessions in addition to the individual patient consultations with the physician within the group setting. The control group consisted of usual care in the majority of studies, however two studies employed enhanced usual care. In the studies by Trento et al. (20, 21, 25), patients attending 7 to 14 quarterly SMAs were compared with a control group of patients also attending between 7 and 14 quarterly usual care consultations with individual education sessions over a 2- to 5-year period. Similarly, Naik et al. (26) compared those attending four SMAs over 3 months with a control group of patients who attended two usual care consultations, each with a 120-min diabetes education session following the consultation. The remaining RCTs did not describe the number, frequency, or duration of usual care visits attended by the control groups. The three-arm study by Schillinger et al. (19) compared the SMA intervention with a usual care group and with a group receiving automated telephone self-management support and nurse follow up. Sample characteristics varied across the RCTs. African-Americans made up >70% of the sample in the studies by Clancy et al. (22, 27), while Gutierrez et al. (23) and Rygg et al. (24) included 100% Hispanics or White Norwegians, respectively. Naik et al. (26) included older Veterans (aged 50–90 years) of which 31% were African-American, and Schillinger et al. (19) included a diverse population of low-income/low-literacy Asians, African-Americans, White Latinos and White non-Latinos, and others (Table 1, C). Over half the studies (19, 22, 23, 26, 27) specified an elevated HbA1c (>7 to >8.5%) as an inclusion criteria, while the remaining had no specific HbA1c requirement (20, 21, 24, 25).

### Clinical Outcomes

Clinical outcomes measured included weight, BMI, FBG, HbA1c, lipid profiles, blood pressure, serum creatinine, and albuminuria (Table 1, C). Three trials assessed both body weight and BMI (20, 21, 24, 25) and one examined BMI only (19). Only the RCTs by Trento et al. (20, 21, 25) found a difference in weight or BMI between SMA and control groups after 2, 4, and 5 years of quarterly SMAs. In these RCTs, no changes in weight were observed after two years; however, at four- and five- years follow up, change in body weight was −1.6 and −3.5 kg in the SMA group compared to −0.24 and +2.0 kg in the control group, respectively. Similarly, BMI reduced by −0.6 kg/m^2^ in the SMA group compared to +0.6 kg/m^2^ in the control group in the four-year trial (21), with trends for reduced BMI also observed in the 2-year (25) and 5-year (20) trials. In contrast, Rygg et al. (24) found no differences in weight or BMI after biweekly or triweekly SMAs for 6–9 weeks and Schillinger et al. (19) reported no change in BMI after monthly SMAs for 9 months. Discrepant findings may be due to the different sample sizes since the only RCT which reported a significant reduction in both weight and BMI in the SMA group was the largest trial with 581 participants (21). It also appears that studies with longer durations had more favorable effects on anthropometric outcomes. This is likely because a prolonged follow up would allow more time for patients to implement the weight-loss advice received through SMAs and for investigators to observe changes in these outcomes.

Fasting blood glucose was measured in the two RCTs by Trento et al. (20, 21, 25), one of which reported no differences between groups at 2-years (25) and 5-years (20) follow up, while the second (21) reported a significantly lower FBG level in the SMA group after four years of quarterly SMAs. Again, this is likely due to the larger sample size (n = 518) in the latter study and greater statistical power to observe effects. HbA1c was measured in all eight RCTs, four of which reported a reduced HbA1c after 12 months to 5 years of SMAs (20, 21, 23, 25, 26). One of these trials found that HbA1c remained stable in the SMA group (7.4% at baseline; 7.5% at 2 years; 7.3% at 5 years), but worsened in the control group, despite the control group receiving usual care plus individual education sessions (7.4 to 8.3 to 9.0%, respectively) (20, 25). Similarly, in the second trial which was the large four-year RCT by Trento et al. (21), HbA1c at follow up was 1.5% lower in the SMA group compared to controls. The study by Gutierrez et al. (23) in a Hispanic population reported a 1.2% reduction in HbA1c in the SMA group after 17 months, compared to a 0.7% reduction in controls, while the study in older Veterans by Naik et al. (26) found that HbA1c at 12 months follow up was 0.7% lower in the SMA group compared to the control group who received enhanced usual care with two diabetes education sessions. In the remaining four trials (19, 22, 24, 27), no significant differences in HbA1c were seen between SMA and control groups after 6–12 months. Disparate findings may be explained by the substantial heterogeneity in study design, including in patient characteristics, the durations and frequencies of SMAs, and lack of standardization of the interventions provided. Importantly, SMAs appeared to be less effective in studies which were <12 months in duration, or where the population included diverse ethnic groups or non-English speakers (19) or was limited to low-income and low-literacy populations (19, 27). Consistent with findings from the retrospective and prospective studies discussed above, this highlights the need for longer follow ups to observe benefits of SMAs, and suggests that perhaps cultural or socioeconomic factors may influence the extent by which SMAs can improve clinical outcomes.

Blood pressure was measured in three trials (19, 21, 24) but only the large four-year RCT by Trento et al. (21) found a significant difference between SMA and control groups in both SBP (138.0 versus 143.6 mmHg, respectively) and DBP (79.1 versus 80.6 mmHg, respectively). Lack of effects observed in the other two trials may be due to the shorter durations (≤12 months versus 4 years) and fewer number of SMAs attended (<5 versus 14 SMAs).

Five of the eight studies measured serum lipids, including total cholesterol (5 RCTs), HDL (5 RCTs), LDL (3 RCTs), and triglycerides (4 RCTs). Of these, three trials (22, 24, 27) reported no differences in lipid profiles between those who attended 3–12 SMAs over 6–12 months compared to controls. Conversely, Trento et al. (25) found that HDL increased by +0.2 mmol/L in patients who attended seven to eight SMAs quarterly for two years compared to those attending usual care plus individual educational sessions; however there were no differences in total or HDL cholesterol or triglycerides at the 5-year follow up of the same patients (20). In the larger study by Trento et al. (21) (n = 518), patients who attended a total of 14 SMAs quarterly over four years had increased HDL at follow up compared to controls (+0.1 versus −0.01 mmol/L), as well as reduced total cholesterol (−0.7 versus +0.1 mmol/L), LDL (−0.6 versus −0.01 mmol/L), and triglycerides (−0.4 versus +0.1 mmol/L). Differences in sample sizes, study durations, and frequency of SMAs may, in part, explain the disparate findings. Similar to findings from observational studies, it appears that improvements in some or all serum lipids in these RCTs were more frequently reported in larger studies, with more frequent SMAs (7–14 SMAs) attended over longer durations (>12 months) (21, 25).

Other clinical outcomes measured included creatinine (4 RCTs) and albuminuria (1 RCT). Again, only the large four-year trial by Trento et al. (21), found a significant increase in creatinine in the control group (+8.8 mmol/L) with no change in the SMA group (+0.9 mmol/L). There were no differences in albuminuria between groups at two or 5 years follow up in the single RCT which measured this outcome (20, 25). Further studies are needed to confirm whether attending SMAs can improve these outcomes compared to usual care.

### Other Outcomes

Other outcomes measured in these studies included cost-effectiveness of SMAs, adherence to American Diabetes Association (ADA) guidelines, quality of life (QoL), diabetes knowledge scores, and diabetes self-efficacy. Two studies by Clancy et al. (22, 28) compared the costs of SMAs to usual care [cost-analysis for 2003 study (22) reported in a separate article (29)]. The 2003 study (22, 29) found significantly higher total costs (inpatient, outpatient, and ED costs) for SMAs compared with usual care (USD $2,886 versus $1,490 per patient over 6 months). These increased costs may have resulted from ‘patient activation’, whereby SMAs alerted patients to the importance of certain aspects of care and increased their service-utilization to ‘catch up’ to care which had previously been neglected (29). It is also likely that the six month study duration provided insufficient time to observe benefits of SMAs in improving self-care and reducing costs in the long term. This is supported by the later study by Clancy et al. (28), where the same costs after one year were significantly lower in the SMA group (USD $5,869 versus $8,412 per patient) compared to controls. Similarly, the Trento et al. study which reported outcomes at two (25) and 5 years (20) follow up also published a separate cost-analysis (30) at 4-year follow-up in the same patients. Here, although the SMA group had slightly higher direct and indirect costs; authors concluded that SMAs were cost-effective as only USD $2.12 was required for an extra point gained on the QoL score (30). Overall, costs are an important consideration when evaluating the feasibility of SMAs, and further studies elucidating the cost-effectiveness of SMAs are warranted.

In the two studies (14, 28) which measured adherence to ADA guidelines, both reported an increased adherence to the recommended standards of care after monthly SMAs for 6–12 months compared to controls. While SMAs may be beneficial for promoting adherence, further studies are needed to confirm this.

Four RCTs (20, 21, 23–25) measured QoL and diabetes-related knowledge. All but one (24) reported improved QoL scores in SMA groups compared to controls, following attendance to >7–8 SMAs for 1.5–5 years. In the one study which found no difference in QoL between groups (24), patients attended only three SMAs over a 6- to 9-week period, which may have been an insufficient number of SMAs and/or inadequate time to observe changes in QoL in these patients. Nevertheless, all four studies (20, 21, 23–25) reported improved diabetes knowledge in the SMA group at study-end, compared to controls, likely due to the educational component included in SMAs.

Regarding diabetes self-efficacy (a tool which evaluates a person’s ability to perform certain diabetes-related management tasks), this was measured in two RCTs (19, 26), both of which found no improvement in those who attended four SMAs over 9–12 months compared to controls. These studies were restricted to older Veterans (26) or low-income low-literacy groups (19), which limits the generalizability of their findings. Additional psychosocial and behavioral outcomes were assessed including health behaviors, problem areas in diabetes, diabetes treatment satisfaction, problem-solving ability, trust in physician, and patient care assessments. Outcomes for these measures were highly variable across the studies, primarily due to the lack of standardization and use of different tools and scoring methods, as well as the different types of SMA interventions delivered, where some included goal setting, interactive learning, and problem-solving activities, while others did not. Importantly, many variables were reported only in individual studies, thus limiting the ability to compare findings across studies.

In summary, it appears that clinical outcomes from RCTs are mixed, as with the previous studies reviewed. Cost comparisons are also unclear, although cost-effectiveness may be more in favor of SMAs and further good-quality studies are required to clarify this. Behavioral factors such as adherence, QoL, and knowledge appear to be benefits of SMAs; however there is less convincing evidence for other behavioral change.

## Summary and Limitations of the Literature

Overall, studies examining the effectiveness of SMAs in improving clinical outcomes for T2DM patients have produced inconsistent results. Approximately half the studies found beneficial effects on anthropometric and metabolic outcomes (5 of 9 for weight and/or BMI; 2 of 3 for FBG; and 8 of 14 for HbA1c), while a lesser number of studies reported significant effects on blood pressure and lipids (3 of 11 for both). It was apparent however, that studies with larger sample sizes and regular/frequent SMA visits over longer follow up periods (>12 months), were more likely to report beneficial effects on clinical outcomes, and this was consistent across the different study designs.

Our findings align with recent meta-analyses (31–33) of observational studies and randomized and non-randomized trials which examined group visits in cohorts of patients with type 1 or type 2 diabetes, or a mix of both (32). All three meta-analyses reported improved HbA1c (31–33), and one also reported improved SBP in group visits compared to usual care (31), while another reported that weight, FBG, DBP, and some lipids improved, however this was inconsistent across different time-points (33). In subgroup analyses, Odgers-Jewell et al. (33) reported a greater effect on HbA1c in studies with smaller numbers of patients per group session and greater contact time; however significant heterogeneity was observed. In meta-regression by Housden et al. (32), patients who attended group visits for longer periods had better HbA1c outcomes, with a 0.25% reduction for every year of participation. The frequency of group visits did not fully explain the difference in effect size, indicating that duration of treatment had a greater effect on HbA1c than the number of visits attended per year (32). However, these meta-analyses differ from the present review in that they all included studies where the group visit teams did not necessarily include a physician, and hence these visits did not meet the classical definition of a SMA (31, 32). To date, no systematic reviews or meta-analyses have examined the effectiveness of SMAs (which by definition must include a physician) in the management of T2DM. Here, we address this knowledge gap by reviewing studies of physician based SMAs, where we found that more regular/frequent SMA visits over longer durations appeared to be more beneficial for improving clinical outcomes compared to usual care in T2DM patients.

Our review also highlights key limitations and substantial heterogeneity in the literature, particularly for non-clinical outcomes including cost-effectiveness, adherence to guidelines, diabetes knowledge and self-management, and health behaviors. For instance, some studies (10, 29) reported increased costs associated with greater health-care utilization (total, ED, inpatient, or outpatient visits), while others reported reduced costs (28), particularly when improved QoL scores were factored in (30), or when decreased medication was used as a proxy for estimating health-care costs (18). Lack of consistency in the methods used to determine cost reductions associated with SMAs hinders our ability to draw valid conclusions regarding their cost-effectiveness in comparison to usual care. To further complicate the matter, what constitutes usual care and the number and frequency of visits in the usual care control groups was defined inconsistently or not at all in many of the studies, which is a key factor needed to make accurate comparisons regarding whether the SMA model is superior to standard care.

For other outcomes, such as adherence to guidelines, QoL, and diabetes knowledge, most studies reported that SMAs were more effective in improving these outcomes compared to usual care. However, interpretation of these findings is limited by several factors. First, whether these improvements translated into better self-management and improved clinical outcomes was not captured in the current literature. Given that none of the studies examined maintenance of effects after the intervention ended, it remains unclear whether clinical or behavioral outcomes of SMAs are sustainable in the long term.

Second, the complex and multi-faceted interventions delivered and different tools used to measure these outcomes make it difficult to compare findings across studies. Consequently, it was not possible to ascertain which, if any, interventions within the SMAs (e.g., improved peer support or improved self-management) were most effective and for whom. Most studies also did not describe the components of the intervention in sufficient detail for replication, particularly the content of the group education sessions. Comparisons of SMA interventions versus enhanced usual care, which include individual or group educational sessions, also warrant further investigation.

Third, many studies tended to focus on very narrow population groups, such as Veterans, refugees, rural or minority ethnic groups, or low-income low-literacy populations. While targeting these high-need and high-risk groups is important, it makes it difficult to generalize results on a broader population-scale. Large-scale studies would help to clarify the practicality of SMAs on a population level and may also identify subgroups of patients which would benefit more or less from SMAs. Whether SMAs may be effective in engaging those patients who would not otherwise engage with usual care also remains to be established.

Moreover, limited data were reported on patient or provider satisfaction, and these elements are critical for successful implementation of SMAs. As yet, it remains unclear whether certain groups would be more accepting of SMAs than others. It is hypothesized that certain individuals, such as independent or self-motivated patients may find the disclosure of illness and the group nature of SMAs stressful (34). Certain cultural groups, particularly those in tight-knit communities, may require greater anonymity in the clinical encounter to prevent community-based stigma, and hence may be opposed to the SMA model (34). This may further vary according to SMA type, thus additional studies are needed to clarify what works and for whom.

Finally, most studies were not conducted in “real world” settings, with most taking place in academic, government, or vertically integrated systems such as Veteran Administrations, all of which tend to have very high quality of care (31). These settings were likely chosen because chronic care redesign interventions are difficult to implement in independent fee-for-service clinics, primarily due to lack of research interest or lack of financial benefits (31). Although it is unclear whether the settings used in these studies influenced their results, caution should be taken when considering using these findings to guide SMA implementation outside such systems.

Overall, our review highlights that there are far more gaps in the literature than there are definitive results regarding the benefits of SMAs in the management of T2DM. Currently, the elements of SMAs that are important for potency, effectiveness, or generalizability, or which are critical in predicting improved outcomes, remain unknown. The nature and variations of the SMA process are likely to always create some confusion and difficulties in interpreting the evidence. While more structured studies may decrease this confusion, there is an immediate need for more Proof of Concept work to show the potential value of such an approach in clinical practice, as well as to provide direction for the most appropriate ways in which these may be offered. Without further studies which elucidate the most effective SMA interventions and the populations which would benefit most, implementation of these SMAs for the management of T2DM will be based more on reasoned judgment than on evidence-based decision making.

## Conclusion and Future Directions

Type 2 diabetes mellitus is a complex chronic disease often accompanied by complications and comorbidities (1). Managing T2DM *via* an integrated model of care such as SMAs may provide a practical solution for relieving the increasing time and cost pressures for both physicians and the broader health system. Designed to meet the clinical, educational, and psychosocial needs of patients, SMAs may be more effective and more popular among both patients and providers than usual care in engaging patients and promoting education and self-care, with potential long-term cost and health benefits.

Our review suggests that SMAs may improve intermediate clinical outcomes for T2DM, such as HbA1c, as well as some behavioral outcomes such as diabetes knowledge and adherence to guidelines, but only when SMAs are attended regularly over longer periods of time. While it is expected that any model of care delivered regularly over time would likely improve clinical outcomes, SMAs have the potential to achieve this while saving costs and relieving some of the current time and resource demands on physicians and the wider health-care system. However, the effectiveness of SMAs in this regard is yet to be established. Collectively, our findings suggest the need for a modified form of SMAs which offers a structured educational program together with the benefits of a medical consultation, more appropriately called a “Programmed Shared Medical Appointment” (PSMA). We are currently testing Proof of Concept of PSMAs for weight control in Australia in a project due to be completed in 2018.

In the meantime, definitive conclusions cannot be drawn regarding the most appropriate patients or settings for a diabetes SMA, nor on the SMA components which may be particularly successful. Further studies are needed to address the knowledge gaps identified in this review, with particular emphasis on: (i) studies with standardized tools to determine the most effective SMA frequencies, durations, and interventions and to closely monitor behaviors; (ii) large-scale RCTs of community-based populations to clarify the broader applicability of SMAs and their short- and long-term clinical and cost outcomes; and (iii) quasi-experimental implementation studies which assess patient- and provider-centered outcomes to determine the “real-world” impact of SMAs and to identify potential barriers to their successful integration into routine care. Only once such studies are widely available will we be able to determine the most effective and economical form of SMAs, as well as the future role of this model of care and its potential for improving outcomes for patients with T2DM.

## Author Contributions

KM and AM screened, reviewed, and interpreted the literature, contributed to the design of the work, and drafted the manuscript. GE and GS contributed to the design of the work and interpretation of the literature and to drafting and editing the manuscript. BC was responsible for the conception and design of the work, interpreted the literature, and contributed to writing and editing the manuscript, and is the guarantor of the review. All authors made substantial intellectual contributions to design and interpretation, drafting and edited the manuscript, and approved the final version, and all authors are responsible for the accuracy and integrity of the manuscript content.

## Conflict of Interest Statement

The authors declare no conflicts of interest and that the research was conducted in the absence of any commercial or financial relationships that could be construed as a potential conflict of interest.
